# Effects of Aging on Arm Swing during Gait: The Role of Gait Speed and Dual Tasking

**DOI:** 10.1371/journal.pone.0136043

**Published:** 2015-08-25

**Authors:** Anat Mirelman, Hagar Bernad-Elazari, Tomer Nobel, Avner Thaler, Agnese Peruzzi, Meir Plotnik, Nir Giladi, Jeffrey M. Hausdorff

**Affiliations:** 1 Center for the study of Movement, Cognition and Mobility, Department of Neurology, Tel Aviv Sourasky Medical Center, Tel Aviv, Israel; 2 School of Healthy Related Professions, Ben Gurion University, Beer Sheba, Israel; 3 Department of Neurology, Tel Aviv Sourasky Medical Center, Tel Aviv, Israel; 4 Information Engineering Unit, POLCOMING Department, Sassari University, Sassari, Italy; 5 The Center of Advanced Technologies in Rehabilitation, Sheba Medical Center, Ramat Gan, Israel; 6 Department of Physical Therapy, Sackler Faculty of Medicine, Tel Aviv University, Tel Aviv, Israel; 7 Sieratzki Chair in Neurology, Sackler Faculty of Medicine, Tel Aviv University, Tel Aviv, Israel; 8 Department of Neurology, Sackler Faculty of Medicine, Tel Aviv University, Tel Aviv, Israel; 9 Sagol School of Neuroscience, Tel Aviv University, Tel Aviv, Israel; University of California, Merced, UNITED STATES

## Abstract

Healthy walking is characterized by pronounced arm swing and axial rotation. Aging effects on gait speed, stride length and stride time variability have been previously reported, however, less is known about aging effects on arm swing and axial rotation and their relationship to age-associated gait changes during usual walking and during more challenging conditions like dual tasking. Sixty healthy adults between the ages of 30–77 were included in this study designed to address this gap. Lightweight body fixed sensors were placed on each wrist and lower back. Participants walked under 3 walking conditions each of 1 minute: 1) comfortable speed, 2) walking while serially subtracting 3’s (Dual Task), 3) walking at fast speed. Aging effects on arm swing amplitude, range, symmetry, jerk and axial rotation amplitude and jerk were compared between decades of age (30–40; 41–50; 51–60; 61–77 years). As expected, older adults walked slower (p = 0.03) and with increased stride variability (p = 0.02). Arm swing amplitude decreased with age under all conditions (p = 0.04). In the oldest group, arm swing decreased during dual task and increased during the fast walking condition (p<0.0001). Similarly, arm swing asymmetry increased during the dual task in the older groups (p<0.004), but not in the younger groups (p = 0.67). Significant differences between groups and within conditions were observed in arm swing jerk (p<0.02), axial rotation amplitude (p<0.02) and axial jerk (p<0.001). Gait speed, arm swing amplitude of the dominant arm, arm swing asymmetry and axial rotation jerk were all independent predictors of age in a multivariate model. These findings suggest that the effects of gait speed and dual tasking on arm swing and axial rotation during walking are altered among healthy older adults. Follow-up work is needed to examine if these effects contribute to reduced stability in aging.

## Introduction

Human walking is characterized by pronounced arm swing and trunk rotation [1–5]. Nonetheless, many questions remain as to the physiological purpose of arm swing and how it changes with aging. One possibility is that arm swing moves in the direction opposite to the lower limb in order to reduce the angular momentum of the body and conserve energy [1–5]. Energy consumption during gait may be reduced by arm swing, due to the reduction of the ground reaction moment during the impact of the foot on the ground [6,7]. This possibility supports the notion that arm swing is actively controlled during gait. Conversely, other studies posit that the arms act as passive dampers [1,8]. Recent studies suggest that arm swing improves stability [9,10], especially in response to perturbations [1,4,11] and this also enhances energy efficiency [1]. The pendulum-like motion of the arms is not a necessary condition for walking [10] and little is known about the effects of aging on arm swing.

The effects of aging on gait have been extensively reported. These include, for example, a reduction in gait speed and an increase in gait variability in older adults and the exaggerated impact of cognitive function and dual tasking abilities on gait quality, variability, asymmetry and fall risk in older adults [12–15]. In healthy adults, arm swing amplitude increases with gait speed [16,17] and is reduced during cognitive loading [18–20]. This reduction in arm swing may, perhaps, increase the risk of falls, especially in older adults. Plate et al. [20] reported that healthy older adults swing their arms more than younger adults during treadmill walking. This somewhat counter-intuitive finding is contradicted by other studies that have shown lower arm swing amplitudes and less inter-limb coordination in older adults than in young adults [18]. The effects of dual-tasking on arm swing have not been well-studied in older adults.

Thus, the aim of the present study was to investigate the effects of healthy aging on arm swing during over-ground walking by examining the role of gait speed, cognitive loading, and trunk rotation and to provide new insight into the controversial reports in the literature regarding the effects of age on arm swing amplitude and coordination [18,20]. We hypothesized that: a) arm swing amplitude will be lower in older adults than in young adults; b) arm swing in older adults will be more affected by changes in gait speed and cognitive loading than younger adults; and c) that trunk rotation will decrease with aging. In addition, we explored whether arm swing and trunk rotation are independently associated with aging.

## Methods

### Study design and Ethical approval

The study was an exploratory observational study to assess the effects of aging on arm swing and trunk rotation during gait. Ethical approval was obtained from the Tel Aviv Sourasky Medical Center human studies committee. All subjects recruited to the study provided informed written consent prior to participating in this study.

### Participants

Sixty healthy adults between the ages of 30–77 years were recruited to participate in this study. Since our aim was to study the effects of healthy aging, subjects were excluded if they had a neurological or orthopedic condition that affected their gait or arm swing, used a walking aid, had an unstable cardiovascular condition, rheumatic disease, any shoulder disorder that caused limitation of range of motion or pain, suffered from diagnosed major depression (based on DSM IV), or had cognitive decline (Montreal Cognitive Assessment battery scores <26) [21], a diagnosed psychiatric disorder, or were pregnant at the time of the test.

### Protocol

After providing informed written consent, demographic data and medical history were collected. Three small, synchronized lightweight 3-axis accelerometers, gyroscopes and magnetometers (Opal APDM, USA) were adhered to the lower back (level of lumbar vertebrae 4–5 to reference the pelvis) and each wrist using velcro straps to quantify gait, arm swing and axial rotation. The sensors were synchronized to each other and pre-calibrated in reference to the vertical axis in the anatomical position. The participants walked back and forth along a 20-m-long, well-lit corridor for one minute under 3 walking conditions: 1) Preferred, usual-walking speed, 2) Dual task (DT): serially subtracting 3s from a predefined 3 digit number while walking, 3) Fast speed walking: subjects were instructed to “walk as fast and safely as you can”. Testing order was randomized across subjects. Signals were filtered using a low-pass Butterworth filter with a frequency cutoff of 3.5 Hz with a band pass of less than 0.5 dB. Only straight line walking was analyzed.

Mean gait speed and stride length were determined by the average time taken to walk the middle 10 meters. Stride time was determined by automatic identification of the time between two consecutive strikes of the same foot, detected from the vertical axis of the trunk acceleration based on the determination of fiducial points in the gait cycle with a peak detection method, as previously described [22,23]. Stride time variability was determined by calculating the magnitude of the stride-to-stride fluctuations, normalized to each subject's mean stride time (Coefficient of Variation = 100xstandard deviation/mean, after removing the values at the turns) [22,23]. Measures of orientation extracted from the sensors (in quaternions) were converted into Euler angles to extract arm swing amplitude and axial rotation in degrees using Matlab's "quat2angle". Arm swing amplitudes (degrees) were calculated as the range from peak flexion to peak extension taking into account the rotation of one segment relative to a reference segment and followed by an X-Y-Z Euler sequence. The dominant and non-dominant arms were evaluated separately. Asymmetry (ASA) was evaluated based on the method previously reported by Lewek et al (2010). A value of zero indicates complete symmetry in magnitude [24]. ASA was determined as:

$$ASA\;=\;\frac{45^{0}-\text{arctan}\left(\frac{ArmSwing_{more}}{ArmSwing_{less}}\right)}{90^{0}}\times 100\text{\%}$$

Arm swing jerk (the third derivative of acceleration) was calculated to assess smoothness, i.e., lower jerk represents smoother movement [25]. The Phase Coordination Index (PCI) is a measure of the accuracy and consistency of stepping phase generation. Briefly, if the contralateral limb portrays cyclic movement exactly and consistently in the middle of the gait cycle of the other limb, the PCI will have a value of zero; with aging and disease, PCI values increase away from this value [26,27]. PCI was calculated in this study for both the lower extremities (gait PCI) and upper extremities (arm swing PCI) to measure coordination between the arms and legs, respectively.

Axial rotation was assessed to explore the association of the trunk movement to arm swing magnitude. The gyroscope sensor placed on the lower back enabled the quantification of transverse plane angular rotation of the lumbar region around the vertical axis. The magnitude of the rotation was quantified as the total rotation of the trunk during a stride cycle. In addition, axial rotation smoothness (jerk) was assessed as the second derivative of the gyroscope signal in the transverse plane.

The following analysis was applied to quantify the relative timing between arm swing and trunk rotation, as a function of the lag of one relative to the other. First, a Gait Synchronization Index (GSI) [28] was calculated based on the entropy of the phase difference between the two arms (or one arm and the trunk) in order to quantify the phase synchronization between the two signals (0.0 = absence of synchronization, 1.0 = perfect synchronization). The phase lag for the two signals (e.g., arm swing and axial rotation) was determined using the Fourier Transform and compared between groups and across conditions.

### Statistical analysis

In order to assess aging effects in this cross sectional study, we divided the subjects into four subgroups based on age: 30–40, 41–50, 51–60, 61–77 yrs. of age. Outcome variables (e.g., arm swing magnitude, arm swing asymmetry, phase lag) were evaluated for normality and homoscedasticity (within groups). Differences in characteristics between groups were assessed using ANOVA. Repeated Measures Analysis of Variance (RM ANOVA) were applied to test if any of the outcome measures change in the different age groups, with respect to different walking conditions (i.e., usual, fast, dual-task) and to identify any group X condition interactions. When condition effects or between group differences were significant, post-hoc analyses were performed. To assess whether arm swing and axial rotation were independently affected by age, we employed a multivariate regression model controlling for gait speed. B coefficients, 95% Confidence Intervals and p values are presented from the different models. Statistical analyses were performed using SPSS version 21.

## Results

Participant characteristics are presented in Table 1. Gender, height, weight and handedness were similar in all four age groups.

**Table 1:** Participants characteristics (entries are mean±SD or n, %, as indicated).

	30–40 yrs (n = 12)	41–50 yrs (n = 20)	51–60 yrs (n = 14)	61–77 yrs (n = 14)	P-Value
Age (yrs.)	33.3±3.4	45.4±3.6	56.3±3.6	64.6±4.4	-
Gender (n and % Females)	8 (66.7%)	9 (45%)	10 (71%)	10 (71.4%)	0.34
Height (cm)	171.3±7.6	168.6±12.8	168.6±9.6	164.9±7.9	0.47
Weight (kg)	63.4±23.3	77.8±16.3	75.0±17.5	69.7±14.6	0.25
Normal walk velocity (m/s)	1.43±0.15	1.33±0.15	1.26±0.11	1.24±0.18	0.03
Dominant right hand (%)	11 (93%)	16 (80%)	13 (86%)	12 (85.7%)	0.84
Number of subtractions during Dual Task condition	16.2±1.3	15.7±2.4	16.0±2.2	13.1±3.1	0.14

### Gait Measures

Significant between group aging effects were observed in gait speed and stride time variability (see Table 2). As expected, the older adults walked slower and with increased stride time variability, even in the usual walking condition. Group differences were not observed in stride length, but cadence tended to decrease with age (F = 3.14, p = 0.07). These effects were more pronounced in the dual task (DT) condition. For example, gait variability increased by 33.8% in the oldest age group in this condition, as compared to 22.4%, 29.3%, and 26.9% in the three younger groups, respectively (F = 148.4, p = 0.03). Condition effects were also observed in stride length with a significant increase in all groups during the fast walking condition, however, between group effects were not significant.

**Table 2:** Gait, arm swing and axial rotation in the different walking conditions. (mean ± SD).

	Age Groups	30–40 yrs	41–50 yrs	51–60 yrs	61–77 yrs	Condition effect	Interaction effect	Between group effect
**Gait measures**								
**Gait Speed (m/sec)**	**Usual walk**	1.43±0.15	1.33±0.15	1.26±0.11	1.24±0.18	0.0001	0.60	0.03
	**DT walk**	1.47±0.25	1.34±0.24	1.23±0.21	1.20±0.24			
	**Fast walk**	1.75±0.21*	1.72±0.24*	1.60±0.21*	1.61±0.19 *			
**Step length (cm)**	**Usual walk**	70.6±4.6	65.5±8.71	67.7±3.9	66.8±7.0	0.0001	0.59	0.68
	**DT walk**	69.8±8.5	65.9±14.9	65.7±14.48	64.6±12.7			
	**Fast walk**	77.3±12.8	75.8±9.2*	74.3±7.9*	75.1±9.1*			
**Stride time variability (%)**	**Usual walk**	1.32±0.3	1.31±0.4	1.36±0.5	1.68±0.8	0.04	0.71	0.02
	**DT walk**	1.38±0.4	1.40±0.5	1.57±0.8*	1.98±0.6*			
	**Fast walk**	1.26±0.3	1.51±0.3*	1.57±0.4*	1.61±0.2*			
**PCI (%)**	**Usual walk**	2.8±1.1	3.9±1.3	4.3±1.2	4.1±1.9	0.0001	0.30	0.07
	**DT walk**	3.5±1.0	4.6±1.9	5.2±3.6	6.3±2.4*			
	**Fast walk**	2.9±0.7	2.9±0.8	3.0±1.1*	3.4±1.1*			
**Arm swing**								
**Dominant Arm swing amplitude (deg)**	**Usual walk**	53.4±16.6	42.5±13.4	42.2±16.0	35.7±13.6	0.0001	0.21	0.04
	**DT walk**	52.8±17.9	48.6±13.0	47.8±21.9	33.5±11.5			
	**Fast walk**	64.6±19.8*	58.3±11.0*	55.6±14.8*	53.9±15.2*			
**Non-dominant Arm swing amplitude (deg)**	**Usual walk**	49.6±16.1	42.3±15.6	42.0±16.7	43.8±14.6	0.0001	0.59	0.68
	**DT walk**	53.5±12.7	47.8±15.9	46.4±14.6	41.8±9.6			
	**Fast walk**	60.4±14.6*	60.2±15.8*	58.2±12.5*	58.4±9.6*			
**Arm Swing Asymmetry (ASA)**	**Usual walk**	10.1±6.3	9.4±4.6	8.2±3.2	6.4±3.0	0.001	0.15	0.34
	**DT walk**	10.2±7.1	9.3±5.9	10.3±6.2	12.7±7.8*			
	**Fast walk**	6.9±2.8*	6.4±3.1*	6.8±2.9*	8.2±3.7*			
**Arm PCI (%)**	**Usual walk**	1.68±0.8	1.23±0.8	1.09±0.4	1.03±0.4	0.34	0.91	0.08
	**DT walk**	1.61±0.8	1.32±0.6	1.21±0.4*	1.27±0.9*			
	**Fast walk**	1.58±0.8	1.22±0.8	1.04±0.1	0.95±0.6			
**Arm swing Jerk (m/s** ^**3**^ **)**	**Usual walk**	0.08±0.06	0.05±0.03	0.05±0.05	0.04±0.04	0.0001	0.27	0.02
	**DT walk**	0.12±0.04	0.08±0.07	0.05±0.02	0.04±0.04			
	**Fast walk**	0.26±0.07*	0.17±0.09*	0.17±0.13*	0.13±0.08*			
**Axial measures**								
**Axial rotation amplitude (deg)**	**Usual walk**	6.71±2.4	5.54±1.9	5.32±1.9	4.44±1.5	0.0001	0.47	0.02
	**DT walk**	7.43±2.8	6.51±2.4	6.30±2.6	4.11±1.6			
	**Fast walk**	8.21±2.7*	6.94±3.2*	7.28±2.9*	5.2±1.6*			
**Axial rotation Jerk (m/s** ^**3**^ **)**	**Usual walk**	3.03±1.2	2.26±0.8	2.05±1.3	1.15±0.6	0.0001	0.05	0.001
	**DT walk**	2.62±1.8	2.06±0.66	1.79±1.3*	1.58±1.0*			
	**Fast walk**	3.78±1.2	3.12±2.0	2.63±1.7*	1.97±0.7*			

P values represent the RMANOVA analysis including differences within the 3 conditions, between groups and any interaction effect for each parameter.

*Significant within group post hoc analysis difference as compared to usual walking condition

### Arm swing measures

Arm swing amplitude in the dominant arm decreased with age under all conditions (see Table 2). In the non-dominant arm, the amplitude decreased in all groups in the DT condition and increased during the fast condition. Post-hoc analysis revealed significant aging effects only in the DT condition (F = 40.87, p = 0.02). Differences in arm swing asymmetry were not significant between groups (F = 1.16, p = 0.34). Asymmetry differed in the fast condition in all groups as compared to usual walking (F = 8.36, p = 0.001). A trend was observed between groups in PCI (F = 3.20, p = 0.08) with the older groups demonstrating lower arm swing PCI than the younger groups (see Table 2). Post-hoc analysis demonstrated a significant increase in arm swing asymmetry and PCI during the DT condition as compared to the usual walking in the two older age groups (p<0.04 and p<0.01, respectively). Significant differences between groups (F = 3.83, p = 0.02) and within conditions (F = 29.68, p<0.001) were observed in arm swing jerk, a measure of smoothness of movement. Smoothness was higher as age increased across all conditions (see Table 2).

### Axial measures

**Fig 1:**
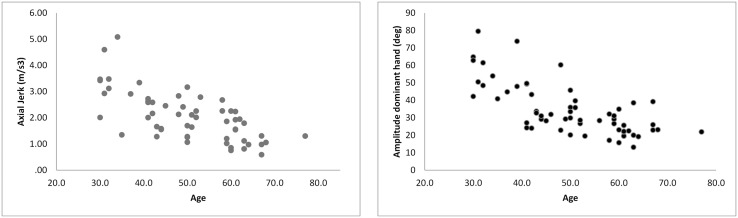
Scatter plots of the effects of age are presented for amplitude of the dominant hand (deg) and axial jerk (m/s3).

Axial rotation amplitude significantly decreased with age (F = 3.31, p = 0.02). Trunk amplitude was significantly lower in the oldest group in all conditions compared to the other groups (F = 18.1, p<0.001). Similarly, axial jerk was significantly lower in the older groups (F = 5.78, p = 0.001). An interaction effect was observed (F = 2.05, p = 0.05) with the two older groups demonstrating an increase in axial rotation jerk in the challenging conditions, as compared to the usual walking conditions while the two younger groups demonstrated a decrease in axial jerk during the DT condition (see Table 2 and see Fig 1).

### Association between age, arm swing and axial measures

Left and right arm swing moved in synchrony in the youngest group under all conditions. In this group, the magnitude of the synchrony was highest during fast walking (0.52±0.07) and lowest during dual tasking (0.47±0.07, F = 7.07, p = 0.02) The magnitude of this arm-arm synchrony decreased with age (F = 4.26, p = 0.009) and there was a significant task effect (F = 28.63, p = 0.001). As expected, the movement of one arm occurred at a delay of ~50% of the gait cycle with respect to the other arm, with no significant aging or task effects on this phase lag.

Arm swing of the dominant arm was synchronized with trunk rotation in all conditions in the youngest group and in most cases (>85%) in the other age groups. The magnitude of the synchrony decreased with aging (F = 7.94, p = 0.001) and there was a significant condition effect (F = 19.01, p = 0.001), i.e., lowest during dual tasking and highest during fast walking. There was approximately a 30% (of the gait cycle) phase lag between arm swing and trunk rotation (i.e., 0.33 sec). This lag was not dependent on age (F = 1.03, p>0.35) and was not related to walking condition (F = 0.61, p>0.55).

Univariate and multivariate regression models were used in order to evaluate the independent contribution of aging to arm swing and axial rotation (see Table 3). The multivariate regression model demonstrated that gait speed, arm swing amplitude of the dominant arm, ASA and axial rotation Jerk were all independent predictors of age accounting for 47.5% of the variance in age.

**Table 3:** Regression models of age, arm swing amplitude and axial rotation.

Dependent measure	Independent measures	Univariate Model	Multivariate Model
**Age**	Gait speed	−34.56, (−54.38–−14.73), **0.001**	−19.56 (−37.88–−1.25), **0.037**
	Arm swing amplitude	−0.36, (−0.49–−0.19), **0.0001**	−0.24, (−0.40–−0.08), **0.004**
	ASA	−0.89, (−1.52–−0.28), **0.005**	−0.67, (−1.19–−0.15), **0.013**
	Axial rotation amplitude	−0.42, (−7.92–−0.84), **0.017**	0.34, (−1.08–1.78), 0.631
	Trunk Jerk	−5.31, (−7.93–−2.68), **0.0001**	−3.69, (−6.52–−0.87), **0.011**
**Arm swing amplitude**	Age	−0.47, (−0.80–−0.14), **0.007**	−0.06, (−0.52–0.39), 0.078
	Gait speed	46.41, (22.88–69.92), **0.0001**	36.94, (4.08–69.81), **0.028**
	Stride variability	−10.05, (−18.19–−1.91), **0.017**	−8.23, (−16.81–0.33), 0.059
	Axial rotation amplitude	2.028, (0.89–3.96), **0.041**	0.76, (−1.69–3.22), **0.043**
	Trunk Jerk	4.52, (1.07–7.98), **0.011**	2.21, (−3.37–7.38), **0.045**
**Axial rotation amplitude**	Age	−0.04, (−0.08–0.003), **0.068**	0.17, (−0.03–0.064), 0.484
	Gait speed	2.71, (−1.12–6.55), 0.162	
	Arm swing amplitude	0.34, (−0.001–0.68), **0.045**	0.01, (−0.02–0.047), 0.413
	ASA	0.25, (−0.92–0.14), 0.432	
	Axial Jerk	1.07, (0.63–1.51), **0.0001**	1.09, (0.57–1.61), **0.0001**

## Discussion

The aim of the present study was to explore the relationships between age, arm swing amplitude and axial rotation movement and to investigate how challenging walking conditions affect these measures. Our findings demonstrate that aging is associated with decreased arm swing amplitude and increased axial rotation jerk during walking. These findings are in line with the known association between aging and decreased gait speed and step length and increased stride time variability [29–32].

The significant negative association between arm swing amplitude and age reflects a decline in arm swing movement during gait with aging. This finding is consistent with the report of Krasovsky et al. [18], but in contrast to that of Plate et al. [20]. The latter group reported that older adults swing their arms more than younger adults while walking on the treadmill. This discrepancy could be related to the difference between over-ground walking which is an internally driven mechanism as opposed to walking on a treadmill which is driven externally by the treadmill speed and has shown to enhance inter-limb coordination [33]. Future work directly comparing arm swing during treadmill and over-ground walking will be helpful for clarifying this issue.

As previously shown [24], we found that amplitude of arm swing while walking correlates with gait speed, resulting in higher amplitudes when walking faster. All subjects, even the older adults, increased their arm swing amplitude in both the dominant and non-dominant hands in the fast walking conditions while differences between groups remained. Arm swing amplitude in the non-dominant hand was significantly increased in all conditions as compared to the dominant hand, reflecting perhaps an active attempt to increase forward momentum while the dominant hand served as the ‘controlled effector’. This is consistent with findings on the control of skillful coordinated movement of the dominant and non-dominant arms, suggesting distinct and different neural control mechanisms for dominant and non-dominant arm movements [34].

Interestingly, group averages of arm swing amplitude were all lower during the DT condition, when subjects tended to walk slower. This effect was larger in the older groups. This finding further supports the association between arm swing and gait speed. In addition, it also suggests that increasing task complexity results in an attempt to decrease degrees of freedom that leads to less movement of both trunk and arms (recall Table 2). The multivariate regression analyses (recall Table 3) suggest that the association between age and arm swing amplitude is mediated by gait speed. While cause and effect cannot be determined here, these findings are all consistent with a relationship between age and arm swing amplitude, with gait speed and dual tasking as potential “confounders”.

Arm swing jerk increased significantly during the fast walking condition reflecting less smooth movements. Intuitively, one might have speculated that increased jerk reflects poorer control. However, already during usual walking conditions, arm swing jerk was highest in the youngest group. Similarly, during fast walking and during dual tasking, this measure was highest in the youngest subjects, twice that seen in the oldest group. In this context, arm swing jerk may reflect flexibility and larger degrees of freedom. As such, one can speculate that the need to maintain dynamic balance during challenging tasks requires the decrease in degrees of freedom that is manifested in a decrease in amplitude of both the arms and trunk and less jerk. Conversely, younger subjects have less difficulty of movement and are not “threatened” by the challenging conditions and, therefore, are more flexible. Indeed, motor control theories suggest that open-loop flexibility seen by increased variability of motor behavior arises from increased sensorimotor control laws optimized for composite cost efficient function thus can reflect health or poor health, depending on its context [35–37].

This interesting finding was also related to the magnitude of axial rotation during fast speed walking. Rotation was lower in the older adults and thus can be seen as a measure of trunk control of movement. Among younger individuals, compared to normal walking, walking with restriction of arm swing induces lower transverse axial rotation [10,38], reflecting the co-dependence of the arms and trunk movement. Moreover, in healthy adults with low back pain, compensation for axial rotation due to pain results in diminished arm swing amplitude and coordination. This change further supports the tight relationship between movements of the trunk and arms while walking [39,40]. These findings are also supported by evidence from studies that reported decreased arm swing amplitude in patients with Parkinson’s disease who have axial rigidity [6,24,41].

We observed that axial rotation and arm swing generally moved synchronously. This attests to the possible co-dependence of these attributes and is consistent with the regression results reported in Table 3 which also support interdependence. Timing was affected by aging and task condition with increased synchronization occurring during the fast condition and decreased synchronization in the DT condition in the older adults group. This finding is consistent with that of Bruijn et al. [16] who reported that timing of thorax rotation changes in different walking speeds, although it maintains a similar pattern and that net thorax acceleration is reflected in arm swing amplitude. These findings support the notion of the passive contribution of neural dynamics to arm swing, although the contribution of active shoulder muscular activity during gait in aging cannot be ruled out based on our findings.

In the early stages of Parkinson’s disease, arm swing asymmetry is more pronounced and is also susceptible to external conditions [24,41]. In our study, we found that arm swing asymmetry was not marked during usual walking in any of the groups. Arm swing amplitudes of the dominant and non-dominant arms were fairly symmetrical with fair bilateral coordination. Bilateral coordination and asymmetry of the lower extremities have been shown to be affected by age [26,27]. Arm swing asymmetry and PCI were lower in older adults than younger adults but were affected by the challenging walking conditions with a significant increase in asymmetry and PCI seen in the older adults groups. These results would suggest that arm swing asymmetry and arm swing PCI are less sensitive to aging than their lower limb parallels. Perhaps, this is a result of the open kinematic chain and the coupling of the lower limbs during gait as compared to upper limbs and the reliance on gravitational support [42]. Further work directly comparing upper and lower asymmetry and coordination could shed additional light on this question.

Asymmetry of the arm swing movement was greatly enhanced by adding a cognitive load of serial subtraction but only in the two older groups. No differences were observed in the performance of the cognitive task between the groups. However, during the dual task condition, values in the two older groups became similar to those of the younger groups, while the dual task condition did not have an impact on arm swing asymmetry in the younger groups (recall Table 2). DT effects have been shown to be dependent on task difficulty [43,44]. Thus, it is possible that the cognitive task was not sufficiently difficult to create a DT effect in younger adults. This possibility is supported by the effects on gait speed: in the young adults, gait speed did not change during DT, while in the older adults, gait speed was reduced.

In summary, the present results support the idea that arm swing changes with healthy aging and is related to gait speed and cognitive loading. This effect is apparently confined to the dominant arm while arm swing asymmetry and bilateral coordination appear to be less sensitive to aging. Interestingly, arm swing jerk and axial rotation jerk were both highly related to aging. Our results suggest that with aging, jerk is reduced in the arms and trunk demonstrating more rigid movement which possibly reflects less flexibility. This decrease in flexibility may be associated with risk of falls and the inability to maintain balance in older age. Additional work is needed to further explore the mechanisms behind this decrease in jerk with aging.
